# Attrition Revisited: Adherence and Retention in a Web-Based Alcohol Trial

**DOI:** 10.2196/jmir.2336

**Published:** 2013-08-30

**Authors:** Elizabeth Murray, Ian R White, Mira Varagunam, Christine Godfrey, Zarnie Khadjesari, Jim McCambridge

**Affiliations:** ^1^e-Health UnitResearch Department of Primary Care and Population HealthUniversity College LondonLondonUnited Kingdom; ^2^Medical Research Council Biostatistics UnitCambridgeUnited Kingdom; ^3^Research Department of Primary Care and Population HealthUniversity College LondonLondonUnited Kingdom; ^4^Department of Health SciencesUniversity of YorkYorkUnited Kingdom; ^5^Department of Social and Environmental Health ResearchLondon School of Hygiene and Tropical MedicineLondonUnited Kingdom

**Keywords:** Internet, eHealth, attrition, adherence, retention, follow-up

## Abstract

**Background:**

Attrition is a noted feature of eHealth interventions and trials. In 2005, Eysenbach published a landmark paper calling for a “science of attrition,” suggesting that the 2 forms of attrition—nonusage attrition (low adherence to the intervention) and dropout attrition (poor retention to follow-up)—may be related and that this potential relationship deserved further study.

**Objective:**

The aim of this paper was to use data from an online alcohol trial to explore Eysenbach’s hypothesis, and to answer 3 research questions: (1) Are adherence and retention related? If so, how, and under which circumstances? (2) Do adherence and retention have similar predictors? Can these predictors adequately explain any relationship between adherence and retention or are there additional, unmeasured predictors impacting on the relationship? (3) If there are additional unmeasured predictors impacting on the relationship, are there data to support Eysenbach’s hypothesis that these are related to overall levels of interest?

**Methods:**

Secondary analysis of data from an online trial of an online intervention to reduce alcohol consumption among heavy drinkers. The 2 outcomes were adherence to the intervention measured by number of log-ins, and retention to the trial measured by provision of follow-up data at 3 months (the primary outcome point). Dependent variables were demographic and alcohol-related data collected at baseline. Predictors of adherence and retention were modeled using logistic regression models.

**Results:**

Data were available on 7932 participants. Adherence and retention were related in a complex fashion. Participants in the intervention group were more likely than those in the control group to log in more than once (42% vs 28%, *P*<.001) and less likely than those in the control group to respond at 3 months (40% vs 49%, *P*<.001). Within each randomized group, participants who logged in more frequently were more likely to respond than those who logged in less frequently. Response rates in the intervention group for those who logged in once, twice, or ≥3 times were 34%, 46%, and 51%, respectively (*P*<.001); response rates in the control group for those who logged in once, twice, or ≥3 times were 44%, 60%, and 67%, respectively (*P*<.001). Relationships between baseline characteristics and adherence and retention were also complex. Where demographic characteristics predicted adherence, they tended also to predict retention. However, characteristics related to alcohol consumption and intention or confidence in reducing alcohol consumption tended to have opposite effects on adherence and retention, with factors that predicted improved adherence tending to predict reduced retention. The complexity of these relationships suggested the existence of an unmeasured confounder.

**Conclusions:**

In this dataset, adherence and retention were related in a complex fashion. We propose a possible explanatory model for these data.

**Trial Registration:**

International Standard Randomized Controlled Trial Number (ISRCTN): 31070347; http://www.controlled-trials.com/ISRCTN31070347 (Archived by WebCite at http://www.webcitation.org/6IEmNnlCn).

## Introduction

### Background

In a landmark paper published in 2005, Eysenbach [1] argued for a “science of attrition” in the field of eHealth research, noting that attrition is a significant issue in many eHealth studies and calling for researchers to report and explore attrition in eHealth studies. He described 2 forms of attrition: nonusage attrition and dropout attrition. Nonusage attrition, also called low adherence, describes the phenomenon of study participants either not using, or not continuing to use, an eHealth intervention. Under research conditions, nonusage leads to an underestimate of the potential efficacy of the intervention, particularly when the intervention was designed to be used repeatedly over time for maximum effect. Dropout attrition, also called loss to follow-up or low retention, refers to study participants not completing follow-up measures. High loss to follow-up decreases the power of a study, and when extreme, makes it hard to interpret the results of a study because there is no way of knowing what effect the intervention had in those who did not provide follow-up data.

To identify papers responding to Eysenbach’s call, we undertook a search of PubMed for papers published between 2005 and the end of 2011 that addressed either form of attrition. The search strategy combined the concepts of Web-based interventions with attrition (either nonusage attrition/adherence or dropout attrition/retention). Of 2581 unique citations, more than 60 papers reported relevant information, including 5 systematic reviews [2-6], 18 trials determining the effects of a specific intervention on either adherence or retention, 3 qualitative studies exploring participant reasons for adherence, 19 studies reporting secondary analyses of data from trials or cohort studies, and a number of studies that used a range of other methodologies. Most (n=45) focused on factors associated with adherence to the intervention, whereas 11 focused on trial retention and 4 looked at the relationship between adherence and retention.

### Adherence to the Intervention (Nonusage Attrition)

Adherence to any specified intervention may be related to characteristics of the intervention, characteristics of the user, or characteristics of the condition addressed by the intervention. Characteristics of the intervention that may improve adherence to the intervention include a strong theoretical foundation [7], perceived personal relevance to the user [8,9], perceived effectiveness [10,11], tailoring [12,13], persuasive technologies [3], credibility [14,15], social networking [16,17], and regular push factors including human support [18-20] and/or periodic prompts either by email or telephone [6]. There is conflicting evidence on adherence and characteristics of the user. Although many researchers have found that women, older people, and well-educated people are more likely to demonstrate adherence to Web-based interventions than males, younger people, and less-educated people [8,9,11,12], others have found no association between adherence and age, gender, or education [21]. As stated by Melville et al [4] in their review of literature exploring the variables associated with adherence to Internet programs for psychological disorders: “Despite the numerous variables explored, evidence on any specific variables that may make an individual more likely to drop out of Internet-based treatment is currently limited.” We were unable to identify data comparing adherence to similar interventions for different health conditions or health behaviors.

### Study Retention (Dropout Attrition)

Improving retention to studies has received less attention in the eHealth literature than improving adherence to interventions. For online questionnaires, the appearance, order, relevance, length, and origin of the questionnaire all seem important [22-24]. Incentives may improve response rates, but may have to have considerable value before having an impact [25]. Providing feedback on questionnaires may improve response rates [26]. There has also been some work characterizing participants who are more likely to drop out from studies, with better response rates reported for people who are white, older, better educated, with good Internet skills [27-31]. Bull et al [32] have had considerable success in improving retention rates in trials of online sexual health promotion. After early difficulties with a trial that only managed 15% retention at follow-up [32], they amended their approach and through a combination of automated electronic and personalized approaches to increase and diversify recruitment, verify participant eligibility and increase retention, achieved a 79% follow-up rate at 1 month [33]. Although eHealth researchers have paid less attention to study retention than to intervention adherence, the general methodological literature has a great deal of information on improving retention, much of which applies to online studies as well as offline ones [34-37].

### Relationship Between Adherence and Retention

In Eysenbach’s original paper, he posited that the 2 forms of attrition were related to one another by a single underlying mechanism—losing interest—and called for empirical studies to test this hypothesis [1].

In a systematic review and meta-analysis of papers published before 2009, Cugelman et al [3] set out to explore 3 outcomes: (1) the overall effect size of online interventions on voluntary behaviors, (2) the impact of various intervention components designed to influence behavior, and (3) the relationship between dose (exposure) of intervention, effect of intervention, and study retention. Thirty-one papers were included in the review. The authors concluded that, despite 1 contradictory correlation, the evidence suggested that intervention adherence was positively correlated with behavioral change. Only 5 (unspecified) studies could be used to assess the relationship between intervention adherence and study retention, but this analysis did reveal a significant positive correlation between them [3]. Subsequently, Couper et al [13] showed that engagement was significantly associated with completion of follow-up data in a randomized controlled trial of alternative versions of an online intervention to promote consumption of fruit and vegetables.

In summary, although many researchers have provided data contributing to a science of attrition, there remain many unanswered questions:

1. Are adherence and retention related? If so, how, and under which circumstances?
2. Do adherence and retention have similar predictors? Can these predictors adequately explain any relationship between adherence and retention or are there additional, unmeasured predictors influencing the relationship?
3. If there are additional unmeasured predictors affecting the relationship, are there data to support Eysenbach’s hypothesis that these are related to overall levels of interest?

This paper addresses these questions through secondary analysis of an Internet-based trial of an online intervention to help heavy drinkers reduce their alcohol consumption.

## Methods

### Ethical Approval

Ethical approval was obtained from University College London ethics committee.

### Study Procedures and Participants

The data reported here were taken from the Down Your Drink (DYD) randomized controlled trial (DYD-RCT; ISRCTN: 31070347), a trial of an online intervention to help hazardous or harmful drinkers reduce their alcohol consumption [38]. Study participants were adults who had browsed the Web and found the DYD home page, which invited them to “find out if you are drinking too much” by completing a brief 3-item screening test, the consumption questions of the Alcohol Use Disorders Identification Test (AUDIT-C) [39]. Those whose score indicated they were drinking at hazardous or harmful levels were invited to take part in the trial (AUDIT-C scores ≥5) [40]. Potential participants were informed that the trial compared 2 websites to see which was more effective in helping users reduce their alcohol consumption. To ensure participants were aware that they were participating in a research study, the informed consent procedures required users to navigate sequentially through 11 pages, provide online consent, create a username and password, and undergo an email validation stage. After their email had been validated, users had to complete baseline data questionnaires, including demographic data, a log of past-week alcohol consumption (the TOT-AL) [41], a 5-item health-related quality of life measure (the EQ-5D) [42], and 1 of 4 randomly allocated secondary outcome measures. Only after all baseline data were completed were participants randomly allocated to either the intervention or the control group. Participants were automatically routed to the website they had been allocated to, so all participants visited their allocated site at least once.

### Intervention and Comparator

The intervention website was a theoretically informed website with multiple interactive features. It contained 3 phases. Phase 1 (“It’s Up to You”) used the principles of motivational interviewing to enable the user to reach a considered decision on whether to change their alcohol consumption. Phase 2 (“Making the Change”) used cognitive behavioral therapy techniques to help users reduce their consumption, whereas Phase 3 (“Keeping on Track”) focused on relapse prevention, a further cognitive behavioral approach. Interactive e-tools, such as the drinking episode diary, provide opportunities for users to reflect on the role alcohol plays in their life and consider alternatives [43]. Users were free to use the intervention website in any way they wanted, but there was an expectation among the developers that users would log on repeatedly to use all elements of the program. The comparator website had a similar look and feel in terms of colors, graphics, and tone. It presented simple information about the harms of excess alcohol consumption, with untailored advice on how to cut down. It had no interactive tools and no drinking diary.

### Follow-Up

The primary outcome point was 3 months after randomization. Data collected at follow-up included past-week alcohol consumption (the primary outcome), the EQ-5D, and the same secondary outcome measure completed at baseline. Data were collected online, with participants sent an email request for follow-up data. The email contained an embedded hotlink that led through to the questionnaires. Up to 3 reminders were sent at 7-day intervals to nonresponders, with the final reminder asking participants to tell us their total past-week alcohol consumption only. This follow-up regime was selected on the basis of our pilot study, exploring different methods of optimizing retention [40].

### Measures of Attrition and Retention

For the purposes of this paper, the 2 outcomes of interest were adherence to the intervention and retention in the trial. Adherence to the intervention was categorized by number of log-ons to the site into 3 groups: users who logged in once only, users who logged in twice, and users who logged in 3 or more times. This categorization was empirically based because the content was highly person-centered so that there were no recommended patterns of use. Retention was treated as a binary variable: participants either did or did not provide follow-up data at 3 months.

The independent variables were the data collected at baseline. Demographic variables were age, gender, educational status (categorized as having vs not having a university degree), marital status (categorized as married/living with partner vs single), having children (categorized as 1 or more vs none), ethnicity (white British or anything else), country of residence (Britain vs rest of the world), and providing offline contact details (provided either address or phone number vs not providing any offline details). Clinical variables were past-week alcohol consumption in units (in which 1 unit is equivalent to 8 g of ethanol), EQ-5D scores, and scores on 2 single-item measures of confidence and intention, both scored from 1 to 5 with 5 indicating the highest level of confidence or intention. For these items, participants were asked, “How confident are you in your ability to reduce your drinking?” and “How strong would you rate your intention to reduce your drinking in the next 3 months?” The EQ-5D scores were obtained from the 5-item questionnaire following the standard procedures [44].

### Statistical Methods

Predictors of adherence and retention were modeled by using logistic regression models with outcomes whether a user (1) logged in twice or more, (2) logged in 3 times or more, and (3) responded at 3 months. To explore the association between adherence and retention, dummy variables for exactly 2 log-ins and 3 or more log-ins were included in model 3.

To select a set of independent variables for all adjusted analyses, we first fitted models 1 to 3 using all the demographic and clinical baseline variables listed previously as independent variables. Past-week alcohol consumption was log-transformed after adding 1 unit/week. For each independent variable, we found the smallest *P* value across models 1 to 3 and we dropped the independent variable with the largest value of this smallest *P* value. To focus on stronger predictors, we repeated this procedure until each independent variable was significant at *P*<.01 in at least 1 of the 3 models. An interaction between TOT-AL and gender was included to allow for women’s typically lower levels of drinking. Goodness of fit was assessed by the Hosmer-Lemeshow test [45]. To facilitate comparisons of the effects of different independent variables, regression coefficients for quantitative independent variables were expressed per 1 standard deviation change. One individual with missing ethnicity at baseline and 2 with missing TOT-AL at baseline were omitted from this analysis.

## Results

### Participants

Data were available on 7932 participants. Demographic and alcohol-related characteristics at baseline are presented in Table 1. The mean age was 38 years, more than half were female, and over half had a university degree. Participants were drinking heavily, with a mean past-week alcohol consumption of 57 units/week. Most participants were intending to reduce their alcohol consumption with a mean score of 3.8 on a scale of 1 to 5; however, they were less confident about their ability to reduce consumption (mean score 2.8).

### Adherence and Retention at Three Months

Of the 7932 participants, 5165 (65%) logged in once only, 1538 (19%) logged in twice, and 1229 (16%) logged in 3 or more times (Table 2). Participants in the intervention group were more likely than those in the control group to log in more than once (42% vs 28%, *P*<.001). These adherence rates were much lower than anticipated for the intervention group, with relatively few users making repeated visits.

Retention also varied by arm. The overall response rate at 3 months was 45% (3528/7932). Participants in the intervention arm were less likely to respond than those in the control arm (40% vs 49%, *P*<.001).

These data appeared to conflict with Eysenbach’s hypothesis of a common factor in adherence and retention because participants in the intervention arm were both more likely to log in to the intervention and less likely to respond to follow-up than those in the control group. However, within each arm, the data were supportive of the Eysenbach hypothesis, with participants who logged in more frequently being more likely to respond at 3 months (Table 2). The overall difference in response rates at 3 months between those with 2 and ≥3 log-ins was smaller than the difference within randomized groups, because this association is confounded by randomized groups. Multivariate analysis confirmed that adherence and retention were positively correlated, with participants who logged in more often being more likely to respond at 3 months (Table 2).

These data suggest that the answer to our first research question (are adherence and retention related?) is yes. In this dataset, adherence and retention are related, but the relationship is not straightforward. Overall, participants who logged in more frequently were more likely to respond at 3 months, but those in the intervention arm were both more likely to log in more than once and less likely to respond than those in the control arm.

### Determinants of Attrition and Retention

Our second research question was “Do adherence and retention have similar determinants? Can these determinants adequately explain any relationship between adherence and retention or are there additional unmeasured determinants impacting on the relationship?”

To address this question, we first explored baseline predictors of adherence and retention (Table 3). Two variables were dropped by the variable selection procedure: marital status (which was correlated with children) and country of residence (which was correlated with ethnicity). The 3 fitted models all had adequate goodness of fit (all *P* values >.05).

Where demographic characteristics were found to predict adherence, they tended to also predict retention and vice versa, although the relationships did not always reach statistical significance. Thus, being older, female, having a university degree, and not having children were all predictive of being more likely to log in more frequently and provide follow-up data at 3 months (Table 3).

In contrast, the relationship between alcohol-related characteristics including past-week consumption, intention to reduce consumption, and level of confidence in the ability to reduce consumption, and adherence or retention went in opposite directions (Table 3). Heavier drinkers, those with higher intention to reduce their drinking, and those with lower confidence in their ability to do so were more likely to log in more often and less likely to respond at 3 months. There was an interaction between amount of alcohol consumed and gender, such that for women, heavier drinking at baseline was associated with greater likelihood of logging on 2 or more times but reduced likelihood of response, but this relationship was not seen for men.

Table 3 also shows that the factor with most impact on adherence and retention was allocation to intervention or control.

These data suggest that the answer to our second research question was that although there was some similarity between the determinants of adherence and the predictors of retention, the relationship between adherence and retention could not be wholly explained by the predictors measured at baseline, suggesting there were additional unmeasured confounders affecting this relationship.

Our third research question was whether the data supported Eysenbach’s hypothesis that participant’s overall level of interest was the factor responsible for any relationship between adherence and retention. As discussed previously, the data pertaining to this question were somewhat contradictory, with adherence and retention positively correlated within each arm of the trial but not across arms because participants in the intervention arm were more likely to adhere but less likely to respond than participants in the control arm.

**Table 1:** Baseline characteristics of the study population (N=7932).

Characteristic	Intervention (n=3970)	Control (n=3962)
Age (years), mean (SD)	37.97 (10.96)	38.29 (10.78)
Gender (female), n (%)	2246 (57)	2299 (58)
Have university degree, n (%)	2067 (52)	2026 (51)
White British, n (%)	3317 (84)	3316 (84)
Have children, n (%)	2052 (52)	2027 (51)
Provided offline address or telephone number, n (%)	1559 (39)	1528 (39)
Past-week alcohol consumption in units^a^, mean (SD)	57.68 (39.62)	56.86 (38.09)
EQ-5D, mean (SD)	0.84 (0.19)	0.84 (0.19)
Confidence^b^, mean (SD)	2.77 (1.16)	2.79 (1.15)
Intention^b^, mean (SD)	3.82 (1.09)	3.85 (1.06)

^a^ 1 unit = 8 g ethanol.

^b^ Confidence and intention scored on a 5-point scale with 1 as the lowest and 5 as the highest.

**Table 2:** Adherence and retention.

Number of log-ins (adherence)	Overall (N=7932)			Intervention (n=3970)			Control (n=3962)		
	n (%)	Responded at 3 months (retention), n (%)	Adjusted OR (95% CI)	n (%)	Responded at 3 months (retention), n (%)	Adjusted OR (95% CI)	n (%)	Responded at 3 months (retention), n (%)	Adjusted OR (95% CI)
1	5165 (65)	2036 (39)	Ref	2324 (59)	792 (34)	Ref	2841 (72)	1244 (44)	Ref
2	1538 (19)	816 (53)	1.79 (1.59, 2.01)^a^	745 (19)	343 (46)	1.79 (1.50, 2.13)^a^	793 (20)	473 (60)	1.88 (1.60, 2.21)^a^
3+	1229 (16)	676 (55)	1.92 (1.68, 2.18)^a^	901 (23)	456 (51)	2.12 (1.80, 2.50)^a^	328 (8)	220 (67)	2.58 (2.02, 3.31)^a^

^a^ *P*<.001

**Table 3:** Baseline predictors of adherence and retention.

Characteristic		Logged in twice OR (95% CI)	*P*	Logged in ≥3 times OR (95% CI)	*P*	Responded at 3 months OR (95%CI)	*P*
**Age** ^a^							
	Per 11-year increase	1.23 (1.16-1.30)	<.001	1.41 (1.31-1.52)	<.001	1.36 (1.29-1.44)	<.001
**Gender (at logtotal + 1 = 3.83)**							
	Females vs males	1.12 (1.02-1.23)	.02	1.12 (0.83-1.28)	.09	1.35 (1.22-1.47)	<.001
**University degree**							
	Degree vs no degree	1.24 (1.13-1.37)	<.001	1.31 (1.15-1.50)	<.001	1.17 (1.07-1.29)	.001
**Ethnicity**							
	White British vs other	0.89 (0.78-1.01)	.08	0.85 (0.72-1.00)	.06	1.24 (1.09-1.41)	.001
**Children**							
	No children vs children	1.12 (1.01-1.25)	.04	1.15 (0.99-1.33)	.06	1.25 (1.12-1.39)	<.001
**Provided address or phone number**							
	Yes vs no	1.1 (0.91-1.11)	.88	0.91 (0.79-1.03)	.14	1.20 (1.09-1.32)	<.001
**Past-week alcohol consumption in units (logtotal + 1)**						
	Per 0.78-unit increase in women^a^	1.13 (1.05-1.21)		1.15 (1.03-1.27)		0.87 (0.81-0.93)	
	Per 0.78-unit increase in men^a^	0.99 (0.93-1.07)		0.97 (0.88-1.07)		0.91 (0.85-0.97)	
**EQ5D**							
	Per 0.19-unit increase^a^	1.02 (0.97-1.07)	.40	0.99 (0.93-1.06)	.80	1.07 (1.02-1.12)	.01
**Confidence**							
	Per 1.15-unit increase^a^	0.92 (0.87-0.97)	.001	0.93 (0.87-1.0)	.04	1.06 (1.01-1.12)	.013
**Intention**							
	Per 1.08-unit increase^a^	1.27 (1.21-1.34)	<.001	1.45 (1.35-1.56)	<.001	0.86 (0.82-0.90)	<.001
**Trial allocation group**							
	Intervention vs control	1.84 (1.68-2.03)	<.001	3.43 (2.99-3.94)	<.001	0.70 (0.64-0.76)	<.001

^a^ Continuous predictors expressed as per 1 SD change.

## Discussion

### Main Findings

Participants in the intervention arm were more likely to use the intervention and less likely to respond to requests for follow-up data at 3 months than those in the control arm. This relationship is likely to be causal because these data were obtained in a RCT in which the only difference between groups was the allocated intervention. We can conclude, therefore, that allocation to the intervention rather than control led to lower levels of follow-up.

Within each trial arm, there was a strong association between logging in more and being more likely to provide follow-up data at 3 months. As expected, the relationship between demographic variables, adherence, and retention tended to be in the same direction, in which factors associated with greater adherence were also associated with greater retention. However, the variables pertaining to alcohol consumption (past-week alcohol consumption, intention to reduce consumption, and confidence in ability to reduce consumption) tended to impact on adherence and retention in opposite directions. Participants who may be more likely to benefit from the intervention (heavier drinkers, drinkers with greater intention to reduce, and those with less confidence in their ability to reduce) made greater use of both intervention and control websites (higher adherence) but were less likely to provide follow-up data.

These data provide some answers to 2 of the 3 research questions posed. In this study, adherence and retention were related. Although there was some similarity between the predictors of adherence and the predictors of retention, the relationship between adherence and retention could not be wholly explained by the predictors measured at baseline, suggesting there were additional unmeasured confounders affecting this relationship.

### Potential Explanatory Model

**Figure 1:**
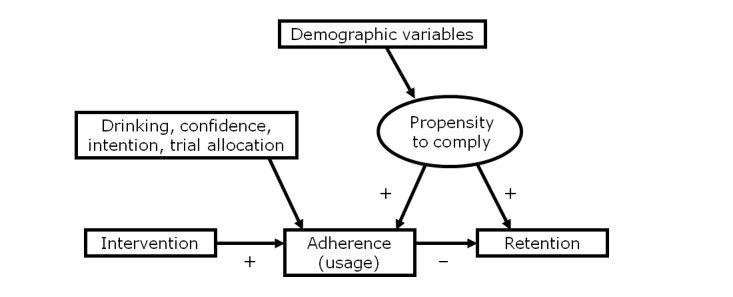
Possible model to explain relationship between adherence and retention.

Our third research question was whether any additional factors influencing the relationship were related to overall levels of interest as postulated by Eysenbach. Our data cannot directly address this question, but 1 possible explanatory model is presented in Figure 1. This model posits that allocation to the active or comparator intervention has a causal relationship with both adherence and retention. Allocation to the active intervention increased adherence to the intervention compared to the control and decreased trial retention. We can speculate that this may reflect at least some people in the intervention arm perceiving the website as helpful and, hence, continuing to use it until they felt they had received an adequate “dose” of intervention. Some participants reported being unable to distinguish between “intervention content” and “trial content” [46]. Therefore, when requests for follow-up data were received, some participants in the intervention arm were more likely to feel they had a sufficient dose and more likely to ignore the requests for follow-up data, even though there was no evidence in the trial that they had changed their behavior any more than participants in the control arm had. In contrast, some people allocated to the control may not have perceived it to be useful because it was not designed to be effective and, thus, continued to have unmet need. Requests for follow-up data may have been seen as an opportunity to monitor and reflect on their alcohol consumption—an opportunity which those in the control group welcomed and were more likely to complete the outcome measures.

The data also suggest that there are user characteristics which influence adherence that act in different ways. We hypothesize that demographic factors are indicative of an unmeasured or latent variable, which we could describe as a “propensity to comply.” Participants with higher propensity to comply are more likely to use the intervention more often and to respond to requests for follow-up data. The propensity to comply may overwhelm the effect of being allocated to the intervention arm on retention. In contrast, alcohol-related factors, including past-week alcohol consumption, intention to reduce consumption, and confidence in one’s ability to reduce consumption, increase use of the intervention and reduce retention.

There are, of course, other possible interpretations of the data. One alternative interpretation is that participants allocated to the active intervention were more frustrated by the gap between their expectations of the intervention and their experience of using it. The active site promised a complete suite of tools to help users make a decision and then act on it, but the low number of log-ons clearly suggested that most users did not use it as planned. It is possible that this disappointment diminished willingness to have contact with the researchers, leading to lower retention. In contrast, the control website only offered straightforward information and, thus, produced less disappointment, leading to greater openness to contact with the researchers, particularly where there were unmet needs as represented by unsuccessful attempts at behavior change.

### Relationship to Previous Literature

Our data build on the available literature in this field, and may help explain some of the contradictory results seen previously. Previous papers have looked for simple relationships between demographic factors, such as age, gender, or education, and either adherence or retention. Our results suggest that a more complex model is required which takes other factors into account. It is also likely that these relationships will vary according to the population and the behavior or condition studied.

### Methodological Strengths and Weaknesses

There are many strengths to these data. They were derived from a large online RCT that had automated randomization ensuring complete concealment of allocation and automated data collection procedures that ensured that all data obtained were of adequate quality for analysis. The large sample size of nearly 8000 participants allowed for highly powered multivariate analyses. The main weaknesses are related to this being a secondary analysis. The initial trial was not designed to address the research questions posed in this paper. We had no a priori definition of adherence and used number of log-ins as the simplest measure of adherence because previous data have shown that number of log-ins and number of pages visited are highly correlated [47]. If there had been a prescribed way of using the intervention we could have looked for adherence to this, but the site was designed to be used differently by different users according to their needs so that participants would make use of the sections or components they found helpful.

### Implications

The data presented here can only be thought of as hypothesis generating. Clearly, further studies specifically designed to test this model and related questions are needed before any firm conclusions can be drawn. However, if confirmed in other studies, these data have implications for both intervention and trial design. Because the biggest predictor of adherence was allocation to the intervention rather than the control arm, researchers need to focus on ensuring that Web-interventions are attractive to the user. The literature suggests that this can best be done by ensuring that the intervention is theoretically informed, has strong perceived personal relevance and effectiveness for users (eg, through tailoring and ensuring credibility), and multiple push factors, both automated (eg, email or short message service text prompts) or human (eg, facilitation or coaching). Previous authors have suggested that it may be possible to identify subgroups of the population who are most likely to adhere [9]. Our data suggest this is unlikely to be fruitful.

The implications for trial design are more challenging to elaborate. If our hypothesis that low retention was related to users in the intervention group feeling that their needs had been met were to be confirmed, this potentially has profound implications for the design of Web-based trials of Web-based interventions.

### Conclusions

In an online RCT of a Web-based intervention to help hazardous and harmful drinkers reduce their alcohol consumption, adherence and retention were related in a complex manner. Some user characteristics, particularly demographic variables, had a positive impact on both adherence and retention, whereas behavioral and related variables increased adherence and reduced retention. We have proposed various possible hypotheses to guide further study.

## Abbreviations

AUDIT-C: Alcohol Use Disorders Identification Test (consumption questions)
DYD: Down Your Drink
OR: odds ratio
RCT: randomized controlled trial
